# Predicting Type 2 Diabetes Remission After Bariatric Surgery: The Role of Homeostatic Model Assessment of Insulin Resistance (Homa-IR), Visceral Adiposity Index (Vai) and Triglyceride-Glucose (TyG) Index

**DOI:** 10.3390/jcm14207273

**Published:** 2025-10-15

**Authors:** Serhat Ocakli, Oktay Banli

**Affiliations:** 1 Department of General Surgery, Faculty of Medicine, Ankara Medipol University, Ankara 06570, Turkey; 2 Obesity Surgery Ankara, Ankara 06680, Turkey; oktaybanli@hotmail.com

**Keywords:** obesity, type 2 diabetes, bariatric surgery, HOMA-IR, triglyceride-glucose index, visceral adiposity index

## Abstract

**Objective:** This study aimed to evaluate the prognostic value of changes in the Homeostatic Model Assessment of Insulin Resistance (HOMA-IR), Visceral Adiposity Index (VAI), and Triglyceride-Glucose (TyG) index in predicting type 2 diabetes mellitus (T2DM) remission following bariatric surgery. **Methods:** This retrospective cohort study analyzed anthropometric, biochemical, and metabolic parameters from 66 T2DM patients who underwent bariatric surgery between 2021 and 2024. Data from the preoperative and 6-month postoperative periods were classified for diabetes remission using American Society for Metabolic and Bariatric Surgery (ASMBS) criteria. **Results:** The mean participant age was 49.9 ± 9.3 years; 72.7% were female. Post-surgery, 51.5% achieved complete remission, 24.25% partial remission, and 24.25% no remission. Only one patient continued insulin, and 83.8% discontinued oral antidiabetics. Significant postoperative improvements were observed in BMI, waist circumference, fasting glucose, HbA1c, triglycerides, HOMA-IR, VAI, and TyG indices, with increased HDL levels (*p* < 0.001). However, preoperative HOMA-IR, VAI, and TyG did not differ significantly across remission groups in univariate analyses. Multivariate logistic regression identified only younger age, higher preoperative BMI, and elevated postprandial insulin as independent predictors of complete remission. Other preoperative biochemical markers were not significantly related to remission outcomes. **Conclusions:** This study indicates that preoperative HOMA-IR, VAI, and TyG have limited standalone value for predicting diabetes remission after bariatric surgery. While they reflect postoperative metabolic improvements, their individual utility in pre-surgical risk stratification is insufficient. More large-scale, prospective studies are needed to determine if these markers could enhance future personalized predictive models.

## 1. Introduction

Obesity and Type 2 Diabetes Mellitus (T2DM) stand out as two major metabolic disorders that threaten global health systems today. According to the World Health Organization, the prevalence of obesity has increased dramatically in recent years, and the incidence of diabetes has accelerated in parallel [1]. Obesity plays a central role in the development of insulin resistance; increased visceral fat tissue triggers inflammatory responses, suppresses pancreatic β-cell functions, and predisposes individuals to hyperglycemia [2]. Indeed, the vast majority of individuals with type 2 diabetes have comorbid obesity, and it is accepted that these two conditions are epidemiologically intertwined [3]. It has been reported that in 2024, approximately 589 million adults worldwide are living with diabetes, and 1.1 billion people are at risk of developing type 2 diabetes [4]. The burden of T2DM attributed to obesity reveals the necessity of focusing not only on glycemic regulation but also on combating obesity in disease control.

In this context, bariatric surgery has become an increasingly preferred method in cases of T2DM that are resistant to lifestyle changes and pharmacological treatments in severely obese individuals. Many studies have shown that bariatric surgeries such as gastric bypass and sleeve gastrectomy (SG) not only provide weight loss but also rapid improvements in glycemic control and metabolic parameters [5,6,7]. For example, bariatric surgery has been shown to be superior to pharmacological therapy in achieving long-term remission and reducing complications in T2DM [8]. In light of these results, bariatric surgery has now taken its place in T2DM treatment algorithms and has been recommended as a treatment option in various international guidelines, referred to as metabolic surgery [9]. The American Diabetes Association (ADA) recommends considering metabolic surgery in all T2DM patients with BMI ≥ 35 kg/m^2^ in its 2023 guideline; it also offers surgery as a potential option for individuals whose BMI is in the range of 30–34.9 and cannot be controlled with traditional treatments [10]. Although high remission rates are achieved after gastric bypass, the multicenter study by Arterburn et al. suggests that this response varies among individuals and that long-term glycemic control is significantly affected by factors such as disease duration and treatment history [11].

This clinical heterogeneity has raised the need for reliable markers that can predict T2DM remission after bariatric surgery. Clinical scoring systems such as the ABCD and DiaRem score, which are among the existing prediction models, are based on parameters such as age, diabetes duration, HbA1c level, C-peptide level and insulin use [12,13]. Although existing predictive systems provide a practical framework for predicting diabetes remission after bariatric surgery, their inadequate reflection of biological heterogeneity and insensitivity to differences among patient populations limit the generalizability and clinical predictive power of these models [14]. Therefore, it is thought that biomarkers that are more objective, quantitative and reflect changes at the metabolic level should be integrated into clinical decision processes.

The Visceral Adiposity Index (VAI) is a sex-specific mathematical model that incorporates waist circumference, BMI, triglycerides, and HDL cholesterol to assess visceral adiposity and its associated cardiometabolic risk. VAI has demonstrated superior correlation with cardiovascular risk factors compared to traditional anthropometric measures and serves as a surrogate marker for visceral fat dysfunction in metabolic disorders. Parameters such as HOMA-IR (insulin resistance indicator), VAI and TyG (triglyceride-glucose index) have the potential to provide important information in both pre-surgical risk stratification and post-surgical recovery monitoring [15,16,17]. Some studies in the literature have reported that post-surgical decreases in these indices may correlate with diabetes remission [18,19]. However, data clearly demonstrating this relationship are still limited, and more comprehensive evaluations are needed on the prognostic value of these indices.

The aim of this study is to evaluate the role of HOMA-IR, VAI and TyG indices in predicting T2DM remission after bariatric surgery and to investigate the potential of these indices to contribute to clinical practices.

## 2. Material and Method

### 2.1. Study Group

This retrospective cohort study was conducted using the data of a total of 66 patients diagnosed with type 2 diabetes who underwent bariatric surgery between January 2021 and December 2024.

Inclusion criteria were as follows: (1) age ≥ 18 years, (2) established diagnosis of T2DM according to American Diabetes Association criteria for ≥6 months prior to surgery, (3) BMI ≥ 35 kg/m^2^ with T2DM, (4) completion of preoperative medical optimization, and (5) complete preoperative and 6-month postoperative data availability. Exclusion criteria included the following: (1) type 1 diabetes or secondary diabetes, (2) previous bariatric or major abdominal surgery, (3) active malignancy, (4) severe psychiatric disorders affecting compliance, (5) pregnancy, and (6) incomplete follow-up data.

### 2.2. Measurements

The patients’ demographic information, comorbidities, surgical methods, preoperative and postoperative 6-month body mass index (BMI), waist circumference, liver ultrasonography (USG) findings, and biochemical data such as fasting and postprandial glucose, insulin, HbA1c, triglyceride and HDL levels were obtained retrospectively from the patient files. Additionally, HOMA-IR, visceral adiposity index (VAI), and triglyceride-glucose (TyG) index were calculated.

Fasting blood samples were collected after a minimum 12 h overnight fast. Postprandial samples were obtained 2 h after a standardized glucose load or meal. All biochemical analyses were performed using standardized automated analyzers in our institutional laboratory. Glucose levels were measured using the hexokinase method on a Beckman Coulter AU480 analyzer (Beckman Coulter Inc., Brea, CA, USA). Insulin concentrations were determined by chemiluminescent microparticle immunoassay. HbA1c was measured using high-performance liquid chromatography standardized to the Diabetes Control and Complications Trial reference method. Complete blood count parameters were analyzed using a Sysmex XN-550 hematology analyzer (Sysmex Corporation, Kobe, Japan). Lipid profiles, including triglycerides and HDL cholesterol, were measured using enzymatic colorimetric methods on the automated chemistry analyzer. All assays were performed according to manufacturer specifications and underwent regular quality control procedures. Laboratory reference ranges and calibration standards were maintained according to Clinical Laboratory Standards Institute guidelines. Inter-assay and intra-assay coefficients of variation were within acceptable limits for all measured parameters.

HOMA-IR was calculated with the formula [Fasting glucose (mg/dL) × Fasting insulin (mU/L)/405]; TyG index was calculated with the formula [Ln (triglyceride [mg/dL] × glucose [mg/dL]/2)]. The VAI was calculated using gender-specific equations, for males: [WC (cm)/39.68 + (1.88 × BMI)] × (TG (mmol/L)/1.03) × (1.31/HDL (mmol/L)); for females: [WC (cm)/36.58 + 1.89 × (BMI)] × (TG (mmol/L)/0.81) × (1.52/HDL (mmol/L)). Postoperative medication use was recorded according to the explanations in the follow-up notes.

The ABCD score was calculated according to Lee et al. [12]. This scoring system incorporates four preoperative variables: age, BMI, C-peptide levels, and duration of diabetes. Higher ABCD scores indicate greater likelihood of diabetes remission. The DiaRem score was calculated using the algorithm developed by Still et al. [13]. This predictive model includes four preoperative variables: age at surgery, HbA1c level, other diabetes medications, and insulin use. Lower DiaRem scores are associated with higher probability of diabetes remission.

Diabetes remission was defined according to established American Society for Metabolic and Bariatric Surgery (ASMBS) guidelines, which align with American Diabetes Association consensus statements on diabetes remission following metabolic surgery: complete remission was defined as HbA1c < 6.0 and fasting glucose < 100 mg/dL; partial remission was defined as HbA1c 6.0–6.4 and fasting glucose 100–125 mg/dL.

Postoperative six-month evaluations were performed during the patient’s outpatient clinic check-up, and biochemical samples and anthropometric measurements were taken on the same day.

### 2.3. Surgery

All surgeries were performed by the senior author. Surgical technique selection between sleeve gastrectomy and One Anastomosis Gastric Bypass (OAGB) was determined by the multidisciplinary committee based on individual patient characteristics. OAGB was preferred in patients with moderate-to-severe hiatal hernia, reflux esophagitis, binge eating behavior, high sweet consumption, or poorly controlled metabolic status. Sleeve gastrectomy was preferred in patients without significant hiatal hernia, with overeating patterns rather than binge eating, and relatively better metabolic control. Additional factors such as smoking status and the need for ongoing endoscopic surveillance were also considered in decision-making.

### 2.4. Statistical Analysis

Statistical analysis of the data was performed using the IBM SPSS v27.0 (IBM Corp., Armonk, NY, USA) program. The conformity of quantitative data to normal distribution was evaluated with the Shapiro-Wilk test. Variables with normal distribution were presented as mean ± standard deviation, and those without normal distribution were presented as median (1st quartile–3rd quartile). Qualitative data were expressed as percentage and frequency. The Wilcoxon signed-rank test was used for preoperative and postoperative comparisons, and the marginal homogeneity test was used for qualitative data. The Kruskal-Wallis, chi-square and Fisher-Freeman-Halton tests were used for comparisons between remission groups. Pairwise comparisons were made with Bonferroni correction for variables with significant differences. Correlations between parameters were analyzed with the Spearman correlation coefficient. Forward selection multiple logistic regression analysis was used to determine factors independently associated with remission. *p* < 0.05 was considered statistically significant.

## 3. Results

The mean age of the 66 patients included in the study was 49.9 ± 9.3 years, and 72.7% of the cases were women. The most frequently applied surgical procedure was OAGB, with a rate of 51.5%, while sleeve gastrectomy was performed with a rate of 48.5%. There was no insulin use in the preoperative period in 69.7% of the patients. At the 6-month post-surgery check-up, it was observed that only one patient required insulin treatment, and 83.8% of them completely discontinued their oral antidiabetic drugs (OAD). When diabetes remission status was examined, it was found that 51.5% of the patients achieved complete remission, 24.2% achieved partial remission, and 24.2% did not achieve remission (Table 1).

When metabolic parameters obtained preoperatively and postoperatively at 6 months were compared, significant improvements were observed in all patients. Significant decreases were detected in insulin resistance indicators such as body mass index, waist circumference, fasting glucose, HbA1c, triglyceride levels and HOMA-IR. HDL level showed a significant increase (*p* < 0.001). Additionally, a significant decrease was observed in Visceral Adiposity Index (VAI) and TyG index values, which are known to reflect visceral fatness (*p* < 0.001) (Table 2).

The mean age of individuals in the complete remission group was found to be significantly lower (*p* = 0.006). Additionally, the sleeve gastrectomy rate was significantly higher in the complete remission group compared to the other groups (*p* = 0.048). Preoperative insulin use was significantly more common in the group in which remission could not be achieved (*p* < 0.001). Preoperative C-peptide levels were significantly higher in patients achieving complete remission compared to those with partial or no remission. ABCD scores were significantly higher in the complete remission group, while DiaRem scores were significantly lower in patients achieving complete remission (Table 3).

After operations, absolute change in body mass index (*p* = 0.005) and waist circumference (*p* = 0.003) values were higher in the complete remission group compared to the partial remission and non-remission groups. Additionally, the decrease in postprandial insulin levels was statistically significant in this group (*p* = 0.029). In the TyG index, a significant difference was observed between the groups only in the postoperative value (*p* < 0.001); no such difference was found in the preoperative value (Table 4).

According to the results of multiple logistic regression analysis, low age (OR: 0.877; 95% CI: 0.797–0.966; *p* = 0.008), high body mass index (OR: 1.199; 95% CI: 1.053–1.366; *p* = 0.006), and high preoperative postprandial insulin (OR: 1.018; 95% CI: 1.005–1.032; *p* = 0.008) were independently associated with complete remission (Table 5). Other variables included in the analysis—operation type (*p* = 0.478), preoperative insulin use (*p* = 0.484), preoperative fasting glucose (*p* = 0.145), preoperative fasting insulin (*p* = 0.403), preoperative HbA1c (*p* = 0.064), preoperative C-peptide (*p* = 0.922), preoperative ABCD score (*p* = 0.927) and preoperative DiaRem score (*p* = 0.269)—were found to be statistically insignificant (Table 5).

## 4. Discussion

This study showed that although significant improvements in metabolic parameters were observed after bariatric surgery, preoperative levels of HOMA-IR, VAI, and TyG indices had limited value in predicting type 2 diabetes remission. Logistic regression analyses revealed that only younger age, high preoperative body mass index, and high postprandial insulin levels were independently associated with complete remission. These results suggest that there is not always a direct relationship between the biochemical proxy of glycemic improvement and clinical remission and that biomarker-based prediction systems should be evaluated with caution.

HOMA-IR, one of the most widely used indicators of insulin resistance, is a practical and frequently used parameter to evaluate the metabolic status of patients, especially before bariatric surgery. Because HOMA-IR is based on fasting glucose and insulin levels, it is considered a practical reflection of this resistance and is often referenced in intervention planning in the preoperative period. In this study, HOMA-IR values decreased significantly at 6 months compared to preoperative levels, and a significant decrease in general insulin resistance was detected. However, it was observed that preoperative levels of this marker did not create a statistically significant difference in predicting diabetes remission. Different results have been reported in the literature on the relationship of HOMA-IR with diabetes remission. Özmen et al. reported that after SADI-S and OAGB applied to T2DM patients with a BMI of approximately 45 kg/m^2^, HOMA-IR levels decreased from 6.2 to 1.4 in 12 months, and complete remission was achieved with HbA1c < 6.0% in all cases; this decrease coincided with complete remission in all cases [19]. Similarly, Lee et al. reported a significant decrease in insulin resistance and increased rates of achieving glycemic targets after SG and RYGB; however, this decrease did not always parallel complete remission [20].

On the other hand, Behrooznia et al. reported in their long-term follow-up studies that despite improvements in HOMA-IR values, permanent remission could not be achieved in some individuals and suggested that this parameter alone was not sufficient [21]. Similarly, Ekberg et al. reported that low HOMA-IR levels did not significantly increase the probability of maintaining normal HbA1c levels in the second year after bariatric surgery, thus demonstrating that HOMA-IR alone is not sufficient to predict long-term glycemic control [22]. These findings suggest that HOMA-IR is not the only pathophysiological process affecting remission. The reversibility of diabetes is determined not only by the improvement of insulin resistance but also by the interaction of many parameters such as pancreatic β-cell reserve, glucagon release, gastrointestinal hormones and hepatic steatosis. Although HOMA-IR is an effective indicator of insulin resistance, it is not a sufficient predictive tool on its own due to the multifactorial nature of remission.

The TyG index has recently distinguished itself as a simple, reliable and effective indicator of insulin resistance. In this study, a significant decrease in the TyG index was observed after surgery; however, preoperative levels did not differ significantly between remission groups. Interestingly, only postoperative TyG values were significantly lower in the complete remission group. This finding suggests that the TyG index may be effective in reflecting the metabolic response after surgery but is limited in terms of prediction before surgery. Jin et al. reported that the postoperative decrease in the TyG index was significantly associated with T2DM remission and that this parameter may be a valuable biomarker in predicting glycemic improvement after surgery [18]. Tahapary et al. argued that TyG is more sensitive than HOMA-IR in reflecting early changes in insulin sensitivity [23].

The findings in this study revealed that the TyG index showed a significant difference in reflecting glycemic improvement in the postoperative period; however, it was insufficient alone to predict remission in the preoperative evaluation. This result may be largely due to the sample structure, number of patients, and single-center design of this study. Additionally, the fact that important variables such as duration of diabetes and β-cell reserve or hormonal response capacity could not be measured in the preoperative period may have limited the predictive contribution expected from an index such as TyG. Considering the limited compatibility of the findings of this study with the literature, it is thought that TyG may be useful in evaluating metabolic response during the follow-up period, but it should be evaluated carefully and within more comprehensive clinical models in preoperative risk stratification.

Visceral Adiposity Index (VAI) is a parameter that reflects visceral fatness and associated metabolic risk, calculated using waist circumference, body mass index, triglyceride and HDL cholesterol levels [16]. The decrease in visceral fatness after bariatric surgery often parallels the decrease in VAI. In this study, VAI values significantly decreased after surgery; however, like HOMA-IR and TyG, preoperative levels did not show a significant relationship with remission. No significant difference was observed between the VAI and remission groups.

The relationship between VAI and remission is not yet clear in the literature. Voglino et al. reported that there were significant improvements in VAI-related measures after surgery and this reduced cardiometabolic risk [24]. Similarly, Liu et al. stated that preoperative VAI can predict postoperative weight loss and revealed that this index has clinical value not only in metabolic monitoring but also in predicting surgical success [25]. Another study has also reported that diabetes remission rates increased significantly in patients with high preoperative VAI levels [19]. However, these studies have largely focused on Asian populations and include differences in genetics, type of surgery, duration of diabetes, and obesity profile. It was concluded that clinically VAI may indirectly contribute to the glycemic control process; however, it cannot be used as a reliable predictive tool in individualized surgical planning.

In this study, while it was observed that HOMA-IR, TyG and VAI indices, which are widely used in the preoperative period, did not make a significant contribution to predicting type 2 diabetes remission, in multiple logistic regression analysis, only young age, high preoperative body mass index, and high postprandial insulin levels were independently associated with remission. This suggests that although biochemical improvements affecting glycemic control are important, they are not sufficient alone to predict a more complex and multivariate outcome such as clinical remission. Dang et al. stated that simple clinical variables, such as insulin use and age, were more powerful than metabolic markers in predicting remission [26]. Grigorescu et al. emphasized that although HOMA-IR and VAI are associated with post-surgical glycemic improvement, their preoperative predictive ability is limited [27]. This discrepancy may reflect differences in study populations, surgical techniques, follow-up duration, and remission criteria. The heterogeneity in findings across studies underscores the need for standardized approaches to biomarker evaluation in metabolic surgery research. These findings indicate that surgical decisions should be supported by multivariate models and that biomarkers can only gain meaning in this context.

The inclusion of established clinical predictors in our analysis provides important context for evaluating metabolic indices. The ABCD score has demonstrated reliable predictive accuracy for diabetes remission following bariatric surgery, particularly in Asian populations. The DiaRem score has been validated across multiple surgical procedures and populations. While these established scoring systems showed the expected univariate associations with remission status in our cohort, neither C-peptide levels (*p* = 0.922), ABCD scores (*p* = 0.927), nor DiaRem scores (*p* = 0.269) reached statistical significance in multivariate logistic regression analysis. Similarly, preoperative HOMA-IR, VAI, and TyG indices showed limited predictive value both in univariate analysis and when considered alongside other clinical variables. These findings suggest that in our cohort, traditional clinical variables such as younger age, higher BMI, and elevated postprandial insulin were stronger independent predictors of remission than established scoring systems or insulin resistance indices. This may reflect the specific characteristics of our patient population or highlight the complexity of diabetes remission prediction, where established models may not universally apply across different populations and surgical contexts.

### Strengths and Limitations

This study’s strengths include the comprehensive evaluation of multiple metabolic indices within a single cohort, standardized surgical techniques performed by a single surgeon, and the use of established ASMBS criteria for remission definition. The simultaneous assessment of HOMA-IR, VAI, and TyG indices provides a comprehensive metabolic profile rarely examined together in bariatric surgery outcomes research.

This study has certain limitations. The definition of diabetes remission with different criteria in the literature and the variety in follow-up periods limit the comparability of the data. Furthermore, fasting-based indices such as HOMA-IR, VAI and TyG may be affected by medication use, hormonal changes and lifestyle factors in the postoperative period. The relatively small sample size (n = 66) from a single center may limit the generalizability of our findings and the power to detect subtle predictive differences between metabolic indices. Additionally, the non-randomized surgical technique assignment based on patient-specific clinical characteristics may introduce selection bias, limiting our ability to directly compare outcomes between procedures. The 6-month follow-up period, while appropriate for initial remission assessment, may not capture long-term diabetes recurrence patterns that could influence the predictive value of these biomarkers. The universal validity of established cut-off values for VAI and TyG is controversial due to ethnicity, gender and anthropometric differences; for example, it should be noted that recommended cut-off values in Asian populations may not have similar predictive power in other groups. Dynamic factors such as lifestyle adaptation, weight regain and treatment continuity, which are effective in post-surgical diabetes management, were also excluded from the scope of the study. Moreover, the findings are based on 6-month short-term follow-up data; however, long-term, multicenter and prospective studies are needed to evaluate the durability of remission.

## 5. Conclusions

This study revealed that HOMA-IR, VAI and TyG indices reflect post-surgical metabolic improvements in type 2 diabetic patients undergoing bariatric surgery, but their preoperative levels have limited clinical value in predicting diabetes remission. The parameters most strongly associated with complete remission were determined to be young age and high preoperative postprandial insulin and body mass index. These findings show that glycemic remission is shaped not only by metabolic indices but also by the interaction of multiple biological and clinical factors. Although indices such as HOMA-IR, TyG and VAI are thought to be useful biomarkers in postoperative follow-up, they cannot be recommended to be used alone as decision-making parameters in patient selection before surgery.

Future research should focus on the following: (1) multicenter prospective studies with extended follow-up periods to validate these findings, (2) development of composite prediction scores incorporating clinical and biochemical parameters, (3) investigation of genetic polymorphisms affecting metabolic responses to surgery, and (4) assessment of these indices’ utility in predicting long-term complications and weight regain patterns.

## Figures and Tables

**Table 1:** Demographic information, operation type and medication use status for all patients.

Variable	
**Age (years)**	49.9 ± 9.3
**Gender**	
Male	18 (27.3%)
Female	48 (72.7%)
**Comorbidity**	
Hypertension	41 (62.1%)
Thyroid disorders	17 (25.8%)
Hypothyroidism requiring replacement	14 (21.2%)
Hyperthyroidism	2 (3.0%)
Thyroid nodules	1 (1.5%)
Coronary artery disease	2 (3.0%)
Sleep apnea	11 (16.7%)
Asthma	5 (7.6%)
**Operation type**	
Sleeve gastrectomy	32 (48.5%)
OAGB	34 (51.5%)
**Pre-op insulin use**	
No	46 (69.7%)
Yes	20 (30.3%)
**Postop insulin cessation**	
No	1 (5.0%)
Quit but started again	0 (0.0%)
Completely quit	19 (95.0%)
**Insulin cessation time**	
Preoperative	0 (0.0%)
Immediately after surgery	19 (100.0%)
Postop 1–3 months	0 (0.0%)
**Preop OAD use**	
No	4 (6.1%)
Yes	62 (93.9%)
**Postop OAD cessation**	
No	5 (8.1%)
Quit but started again	5 (8.1%)
Completely quit	52 (83.8%)
**OAD cessation time**	
Preoperative	4 (7.0%)
Immediately after surgery	49 (86.0%)
Postop 1–3 months	4 (7.0%)
**Remission**	
No	16 (24.25%)
Partial	16 (24.25%)
Full	34 (51.5%)

While quantitative variables were summarized as mean ± standard deviation according to their compliance with normal distribution, qualitative variables were summarized as frequency (percentage).

**Table 2:** Preoperative and postoperative findings for all patients.

	Preoperative	Postoperative 6th Month	*p*
Body mass index, kg/m^2^	42.8 (38.7–50.2)	32.7 (28.5–36.3)	**<0.001 ^†^**
Waist circumference, cm	134.5 (121–148)	103 (88–112)	**<0001 ^†^**
USG liver steatosis			
Grade 0	6 (9.1%)	27 (40.9%)	**<0.001 ^§^**
Grade 1	15 (22.7%)	37 (56.1%)	
Grade 2	29 (43.9%)	2 (3.0%)	
Grade 3	16 (24.3%)	0 (0.0%)	
Fasting glucose, mg/dL	136 (114–164)	96 (89–118)	**<0.001 ^†^**
Postprandial glucose, mg/dL	159 (117–230)	102 (87.2–134)	**<0.001 ^†^**
Fasting insulin, mU/L	13.4 (9.4–20.1)	7.0 (5.4–9.3)	**<0.001 ^†^**
Postprandial insulin, mU/L	28.7 (16.4–98)	12.9 (8.1–23.1)	**<0.001 ^†^**
HbA1c, %	6.9 (6.2–8.2)	5.7 (5.2–6.0)	**<0.001 ^†^**
Triglyceride, mg/dL	161 (118–205)	117.7 (88–156)	**<0.001 ^†^**
HDL, mg/dL	43.5 (37–49)	48.8 (41.6–57)	**<0.001 ^†^**
HOMA-IR	5.1 (3.4–828)	1.6 (1.2–2.3)	**<0.001 ^†^**
VAI	3.1 (2.3–4.3)	1.9 (1.2–2.6)	**<0.001 ^†^**
TyG index	9.3 (8.9–9.6)	8.6 (8.3–8.9)	**<0.001 ^†^**

While quantitative variables were summarized with median (1st quartile–3rd quartile) according to their compliance with normal distribution, qualitative variables were summarized as frequency (percentage). ^†^ Wilcoxon signed-rank test, ^§^ Marginal homogeneity test. Significant *p*-values are presented in bold.

**Table 3:** Demographic information, operation type and medication use status according to remission status.

	DM Remission			
	No (n = 16)	Partial Remission (n = 16)	Full Remission (n = 34)	*p* (Intergroup)
**Age**	55.9 ± 6.8	49.7 ± 10.6	47.1 ± 8.5 *	**0.006 ^†^**
**Gender**				
Male	4 (25.0%)	5 (31.2%)	9 (26.5%)	0.937 ^¶^
Female	12 (75.0%)	11 (68.8%)	25 (73.5%)	
**Operation type**				
Sleeve gastrectomy	4 (25.0%)	7 (43.8%)	21 (61.8%) *	**0.048 ^§^**
OAGB	12 (75.0%)	9 (56.2%)	13 (38.2%) *	
**Pre-op insulin use**				
No	4 (25.0%)	13 (81.3%) *	29 (85.3%) *	**<0.001** **^¶^**
Yes	12 (75.0%)	3 (18.7%) *	5 (14.7%) *	
**Preop OAD use**				
**No**	1 (6.3%)	0 (0.0%)	3 (8.8%)	0.801 ^¶^
Yes	15 (93.7%)	16 (100.0%)	31 (91.2%)	
**Postop OAD cessation**				
No	2 (13.3%)	2 (12.5%)	1 (3.25%)	**0.016** **^¶^**
Quit but started again	4 (26.7%)	0 (0.0%) *	1 (3.25%) *	
Completely quit	9 (60.0%)	14 (87.5%)	29 (93.5%) *	
**C-peptide**	2.9 ± 1.0	3.8 ± 1.6	4.3 ± 1.6 *	**0.008** **^†^**
**ABCD score**	4 (4–5)	5 (3–7)	6 (5–7) *	**<0.001** **^‡^**
**DiaRem score**	17 (13–19)	6.5 (4–10) *	5 (2–8) *	**<0.001** **^‡^**

While quantitative variables were summarized as mean ± standard deviation according to their compliance with normal distribution, qualitative variables were summarized as frequency (percentage). The term ‘remission’ in diabetes refers to achieving glycemic targets without active glucose-lowering medications, as distinguished from ‘cure,’ which implies permanent resolution. ^†^ One-way analysis of variance (ANOVA), ^§^ Chi-square test, ^¶^ Fisher-Freeman-Halton test, ^‡^ Kruskal Wallis test, N/A Not Applicable, * “No remission” different from the group. Significant *p*-values are presented in bold.

**Table 4:** Preoperative and postoperative findings according to remission status.

	DM Remission			
	No (n = 16)	Partial Remission (n = 16)	Full Remission (n = 34)	*p* (Intergroup)
**Body mass index, kg/m^2^**				
Preoperative	41.7 (39.2–47.8)	38.6 (35.5–42.2)	46.6 (39.4–51.9) ^#^	**0.005 ^‡^**
Postop 6. month	32.3 (29.6–37.3)	29.0 (26.8–32.4)	34.5 (29.6–37.5) ^#^	**0.039 ^‡^**
p (intergroup)	**<0.001 ^†^**	**<0.001 ^†^**	**<0.001 ^†^**	
Change ^(a)^	−10.4 (−12.7–−7.9)	−9.8 (−10.3–−8.2)	−12.6 (−15.6–−9.8) ^#^	**0.005 ^‡^**
**Waist circumference, cm**				
Preoperative	128 (122–144)	130 (113–140.5)	144.5 (127–156)	0.051 ^‡^
Postop 6. month	102 (89–115.5)	101 (85–108)	106 (92–121)	0.344 ^‡^
p (intergroup)	**<0.001 ^†^**	**<0.001 ^†^**	**<0.001 ^†^**	
Change ^(a)^	−27.5 (−37–−25)	−30.5 (−35.5–−23.5)	−38 (−47–−31) *^,#^	**0.003 ^‡^**
**Fasting glucose, mg/dL**				
Preoperative	173 (153.5–233)	131 (107–165.5) *	131 (112–146) *	**0.002 ^‡^**
Postop 6. month	133 (119–148)	103.5 (102–113)	90.5 (81–94) *^,#^	**<0.001 ^‡^**
p (intergroup)	**0.011 ^†^**	**0.002 ^†^**	**<0.001 ^†^**	
Change ^(a)^	−47.5 (−103.2–−15)	−31.5 (−60–−4)	−38.5 (−65–−21)	0.435 ^‡^
**Postprandial glucose, mg/dL**				
Preoperative	212 (146–325)	125 (103–186)	141 (117–205.85)	0.086 ^‡^
Postop 6. month	156 (134–202)	94 (87.2–113) *	97 (82–104) *	**<0.001 ^‡^**
p (intergroup)	0.158 ^†^	**0.038 ^†^**	**<0.001 ^†^**	
Change ^(a)^	−14.5 (−191–18)	−21 (−78.8–1.1)	−61 (−139–−24)	0.138 ^‡^
**Fasting insulin, mU/L**				
Preoperative	12.4 (10.4–15.5)	11.1 (6.0–16.1)	18.0 (10.1–29.7) ^#^	**0.026 ^‡^**
Postop 6. month	7.4 (3.6–9.3)	6.2 (4.3–8.3)	7.0 (5.7–10)	0.378 ^‡^
p (intergroup)	**0.001 ^†^**	**0.002 ^†^**	**<0.001 ^†^**	
Change ^(a)^	−6.8 (−10.9–−2.5)	−4.8 (−8.4–−2.3)	−8.1 (−18.2–−3.7)	0.105 ^‡^
**Postprandial insulin, mU/L**				
Preoperative	19.1 (11.6–46)	17.6 (13.6–28.2)	47.7 (22–162) ^#^	**0.015 ^‡^**
Postop 6. month	11.9 (7.2–22.3)	9.0 (7.6–14.0)	15.7 (8.4–25.2)	0.312 ^‡^
p (intergroup)	0.093 ^†^	**0.028 ^†^**	**<0.001 ^†^**	
Change ^(a)^	−11.8 (−35.0–−3.6)	−4.3 (−14.4–−0.01)	−33.2 (−116.1–−14.1) ^#^	**0.029 ^‡^**
**HbA1c, %**				
Preoperative	8.5 (7.5–10.1)	6.7 (6–7.9) *	6.8 (5.9–7.2) *	**<0.001 ^‡^**
Postop 6. month	6.8 (6.6–7.2)	5.7 (5.1–5.9) *	5.4 (5.1–5.6) *	**<0.001 ^‡^**
p (intergroup)	**<0.001 ^†^**	**<0.001 ^†^**	**<0.001 ^†^**	
Change ^(a)^	−1.3 (−2.8–−0.4)	−1.5 (−1.9–−0.5)	−1.3 (−1.7–−0.7)	0.984 ^‡^
**Triglyceride, mg/dL**				
Preoperative	151 (131.5–291.5)	157 (109.5–210.5)	166 (119–196)	0.849 ^‡^
Postop 6. month	132 (108–179.5)	116 (87–155.5)	110 (83–140)	0.128 ^‡^
p (intergroup)	**0.023 ^†^**	**0.005 ^†^**	**<0.001 ^†^**	
Change ^(a)^	−45 (−103.5–−2)	−44 (−102.5–−6)	−39.7 (−86.4–−16)	0.982 ^‡^
**HDL, mg/dL**				
Preoperative	45 (34.5–48.5)	41.4 (36.6–49.7)	43.0 (38–49)	0.861 ^‡^
Postop 6. month	46.9 (38–52.1)	50.5 (42.1–57.5)	49 (40.5–57)	0.511 ^‡^
p (intergroup)	0.155 ^†^	**0.015 ^†^**	**0.011 ^†^**	
Change ^(a)^	5.7 (−3.5–11.2)	7.6 (−0.9–16.1)	2 (−1–10.5)	0.479 ^‡^
**HOMA-IR**				
Preoperative	6.0 (3.9–9.1)	4.7 (1.8–6.5)	5.2 (3.6–9.3)	0.096 ^‡^
Postop 6. month	2.2 (1.2–3.0)	1.68 (1.2–2.0)	1.5 (1.2–2.1)	0.327 ^‡^
p (intergroup)	**<0.001 ^†^**	**0.001 ^†^**	**<0.001 ^†^**	
Change ^(a)^	−3.7 (−6.7–−1.9)	−2.9 (−3.8–−0.6)	−3.6 (−8.0–−1.7)	0.150 ^‡^
**VAI**				
Preoperative	3.5 (2.4–4.9)	3.2 (2.1–4.0)	3.0 (2.5–4.2)	0.704 ^‡^
Postop 6. month	2.3 (1.3–3.2)	1.8 (1.1–2.3)	1.5 (1.2–2.5)	0.129 ^‡^
p (intergroup)	**0.003 ^†^**	**<0.001 ^†^**	**<0.001 ^†^**	
Change ^(a)^	−1.1 (−2.4–−0.9)	−1.3 (−2.5–−0.6)	−1.1 (−2–−0.6)	0.960 ^‡^
**TyG index**				
Preoperative	9.5 (9.3–10.3)	9.2 (8.9–9.7)	9.2 (8.9–9.5)	0.155 ^‡^
Postop 6. month	9.1 (8.8–9.4)	8.7 (8.5–9.0)	8.5 (8.2–8.7) *	**<0.001 ^‡^**
p (intergroup)	**0.015 ^†^**	**<0.001 ^†^**	**<0.001 ^†^**	
Change ^(a)^	−0.5 (−1.0–−0.3)	−0.4 (−1.0–−0.2)	−0.7 (−1.1–−0.3)	0.325 ^‡^

While quantitative variables were summarized with median (1st quartile–3rd quartile) according to their compliance with normal distribution, qualitative variables were summarized as frequency (percentage). ^(a)^ Difference between postoperative and preoperative measurements, negative values indicate a decrease and positive values indicate an increase. The term ‘remission’ in diabetes refers to achieving glycemic targets without active glucose-lowering medications, as distinguished from ‘cure,’ which implies permanent resolution. ^†^ Wilcoxon signed-rank test, ^‡^ Kruskal Wallis test, * “No remission” different from the group, ^#^ “Partial remission” different from the group. Significant *p*-values are presented in bold.

**Table 5:** Factors related to complete remission, multiple logistic regression analysis.

	β Coefficient	Standard Error	*p*	Exp (β) (95% CI)
Age	−0.131	0.049	**0.008**	0.877 (0.797–0.966)
Body mass index, kg/m^2^, Preoperative	0.182	0.066	**0.006**	1.199 (1.053–1.366)
Postprandial insulin, Preoperative	0.018	0.007	**0.008**	1.018 (1.005–1.032)
Constant	−2.354	2.759	0.394	

Nagelkerke R^2^ = 0.519, CI: Confidence interval.

## Data Availability

The data presented in this study are available upon request from the corresponding author due to restrictions regarding patients’ privacy.

## References

[1] Chandrasekaran P., Weiskirchen R. (2024). The Role of Obesity in Type 2 Diabetes Mellitus—An Overview. Int. J. Mol. Sci..

[2] Kahn S.E., Hull R.L., Utzschneider K.M. (2006). Mechanisms linking obesity to insulin resistance and type 2 diabetes. Nature.

[3] Ruze R., Liu T., Zou X., Song J., Chen Y., Xu R., Yin X., Xu Q. (2023). Obesity and type 2 diabetes mellitus: Connections in epidemiology, pathogenesis, and treatments. Front. Endocrinol..

[4] International Diabetes Federation (IDF) (2025). IDF Diabetes Atlas.

[5] Mingrone G., Panunzi S., De Gaetano A., Guidone C., Iaconelli A., Leccesi L., Nanni G., Pomp A., Castagneto M., Ghirlanda G. (2012). Bariatric surgery versus conventional medical therapy for type 2 diabetes. N. Engl. J. Med..

[6] Schauer P.R., Bhatt D.L., Kirwan J.P., Wolski K., Aminian A., Brethauer S.A., Navaneethan S.D., Singh R.P., Pothier C.E., Nissen S.E. (2017). Bariatric Surgery versus Intensive Medical Therapy for Diabetes—5-Year Outcomes. N. Engl. J. Med..

[7] Pories W.J., Swanson M.S., MacDonald K.G., Long S.B., Morris P.G., Brown B.M., Barakat H.A., Deramon R.A., Israel G., Dolezal J.M. (1995). Who would have thought it? An operation proves to be the most effective therapy for adult-onset diabetes mellitus. Ann. Surg..

[8] Yang Y., Miao C., Wang Y., He J. (2024). The long-term effect of bariatric/metabolic surgery versus pharmacologic therapy in type 2 diabetes mellitus patients: A systematic review and meta-analysis. Diabetes Metab. Res. Rev..

[9] Brito J.P., Montori V.M., Davis A.M. (2017). Metabolic Surgery in the Treatment Algorithm for Type 2 Diabetes: A Joint Statement by International Diabetes Organizations. JAMA.

[10] ElSayed N.A., Aleppo G., Aroda V.R., Bannuru R.R., Brown F.M., Bruemmer D., Collins B.S., Hilliard M.E., Isaacs D., Johnson E.L. (2023). 1. Improving Care and Promoting Health in Populations: Standards of Care in Diabetes-2023. Diabetes Care.

[11] Arterburn D.E., Bogart A., Sherwood N.E., Sidney S., Coleman K.J., Haneuse S., O’cOnnor P.J., Theis M.K., Campos G.M., McCulloch D. (2013). A multisite study of long-term remission and relapse of type 2 diabetes mellitus following gastric bypass. Obes. Surg..

[12] Lee W.-J., Hur K.Y., Lakadawala M., Kasama K., Wong S.K., Chen S.-C., Lee Y.-C., Ser K.-H. (2013). Predicting success of metabolic surgery: Age, body mass index, C-peptide, and duration score. Surg. Obes. Relat. Dis..

[13] Still C.D., Wood G.C., Benotti P., Petrick A.T., Gabrielsen J., Strodel W.E., Ibele A., Seiler J., Irving B.A., Celaya M.P. (2014). Preoperative prediction of type 2 diabetes remission after Roux-en-Y gastric bypass surgery: A retrospective cohort study. Lancet Diabetes Endocrinol..

[14] Panunzi S., Carlsson L., De Gaetano A., Peltonen M., Rice T., Sjöström L., Mingrone G., Dixon J.B. (2016). Determinants of Diabetes Remission and Glycemic Control After Bariatric Surgery. Diabetes Care.

[15] Ayranci M., Vatansev H. (2023). Effects Of Bariatric Surgery In Morbid Obese Patients On HOMA-IR And Lipid Profiles. Clin. Nutr. ESPEN.

[16] Amato M.C., Giordano C., Galia M., Criscimanna A., Vitabile S., Midiri M., Galluzzo A., AlkaMeSy Study Group (2010). Visceral Adiposity Index: A reliable indicator of visceral fat function associated with cardiometabolic risk. Diabetes Care.

[17] Guerrero-Romero F., Simental-Mendía L.E., González-Ortiz M., MartínEz-Abundis E., Ramos-Zavala M.G., HernánDez-GonzálEz S.O., Jacques-Camarena O., RodrígUez-Morán M. (2010). The product of triglycerides and glucose, a simple measure of insulin sensitivity. Comparison with the euglycemic-hyperinsulinemic clamp. J. Clin. Endocrinol. Metab..

[18] Jamialahamdi T., Gadde K.M., Nguyen N.T., Kroh M., Sukhorukov V.N., Almahmeed W., Al-Rasadi K., Sahebkar A. (2024). Improvement of Triglyceride-Glucose Index Following Bariatric Surgery: A Systematic Review and Meta-analysis. Obes. Surg..

[19] Ozmen M.M., Guldogan C.E., Gundogdu E. (2020). Changes in HOMA-IR index levels after bariatric surgery: Comparison of Single Anastomosis Duodenal Switch-proximal approach (SADS-p) and One Anastomosis Gastric Bypass-Mini Gastric Bypass (OAGB-MGB). Int. J. Surg..

[20] Lee W.J., Chong K., Ser K.H., Lee Y.C., Chen S.C., Chen J.C., Tsai M.-H., Chuang L.-M. (2011). Gastric bypass vs sleeve gastrectomy for type 2 diabetes mellitus: A randomized controlled trial. Arch. Surg..

[21] Behrooznia Z., Jangjoo A., Qoorchi Moheb Seraj F., Khadem-Rezaiyan M., Zandbaf T., Hassani S. (2023). Diabetic Markers, Five Years after Bariatric Surgery. Middle East J. Dig. Dis..

[22] Ekberg N.R., Falhammar H., Näslund E., Brismar K. (2020). Predictors of normalized HbA1c after gastric bypass surgery in subjects with abnormal glucose levels, a 2-year follow-up study. Sci. Rep..

[23] Tahapary D.L., Pratisthita L.B., Fitri N.A., Marcella C., Wafa S., Kurniawan F., Rizka A., Tarigan T.J.E., Harbuwono D.S., Purnamasari D. (2022). Challenges in the diagnosis of insulin resistance: Focusing on the role of HOMA-IR and Tryglyceride/glucose index. Diabetes Metab. Syndr..

[24] Voglino C., Tirone A., Ciuoli C., Benenati N., Paolini B., Croce F., Gaggelli I., Vuolo M.L., Cuomo R., Grimaldi L. (2020). Cardiovascular Benefits and Lipid Profile Changes 5 Years After Bariatric Surgery: A Comparative Study Between Sleeve Gastrectomy and Roux-en-Y Gastric Bypass. J. Gastrointest. Surg..

[25] Liu F., He J., Zhu Y., Wang H., Feng W., Sun X., Bi Y., Zhu D. (2021). Body Adiposity Index Is Predictive of Weight Loss after Roux-en-Y Gastric Bypass. Ann. Nutr. Metab..

[26] Dang J.T., Sheppard C., Kim D., Switzer N., Shi X., Tian C., de Gara C., Karmali S., Birch D.W. (2019). Predictive factors for diabetes remission after bariatric surgery. Can. J. Surg..

[27] Ghusn W., Ikemiya K., Al Annan K., Acosta A., Abu Dayyeh B.K., Lee E., Spaniolas K., Kendrick M., Higa K., Ma P. (2023). Diabetes Mellitus Remission in Patients with BMI  >  50 kg/m^2^ after Bariatric Surgeries: A Real-World Multi-Centered Study. Obes. Surg..

